# Association Between Treatment-Resistant Sarcoid Myopathy and Inclusion Body Myositis

**DOI:** 10.7759/cureus.6656

**Published:** 2020-01-14

**Authors:** Dario A Marotta, Hassan Kesserwani

**Affiliations:** 1 Department of Research, Alabama College of Osteopathic Medicine, Dothan, USA; 2 Neurology, Flowers Medical Group, Dothan, USA

**Keywords:** sarcoidosis, inclusion body myositis, refractory

## Abstract

The association between sarcoid myopathy and inclusion body myositis is a rare phenomenon that is not well understood. In this case, we present a 46-year-old female with a five-year history of sarcoidosis who became refractory to treatment, experiencing progressive deterioration and muscle wasting. The patient’s distribution of muscle weakness did not follow characteristic patterns of inclusion body myositis. Yet, a subsequent deltoid biopsy revealed diagnostic findings typical of inclusion body myositis. This case report reveals an association between treatment-resistant sarcoid myopathy and the evolution of inclusion body myositis in the absence of characteristic clinical findings.

## Introduction

Sarcoidosis is an idiopathic systemic inflammatory disease characterized by the presence of noncaseating granulomas, which are most commonly found in the lungs, lymph nodes, nervous system, and musculature. These granulomas are believed to be a consequence of a hyperpolarized T-helper (TH1) response to pathogenic tissue antigens arising from a combination of environmental and genetic triggers [1]. Inclusion body myositis (IBM) is an insidious and debilitating disease characterized by distal and proximal myopathy, which, unlike sarcoidosis, has been shown to be unresponsive to contemporary treatment modalities [2]. The pathogenesis of IBM is less understood; however, prevailing theories implicate a combination of autoimmune and degenerative pathways leading to cytotoxic T cell invasion of muscle fibrils and toxic extra-nuclear protein accumulations [3]. Instances of co-existent sarcoidosis and IBM have rarely been documented in the literature [4-7]. This case report describes a 46-year-old female with a history, physical and muscle biopsy consistent with coexisting sarcoid myopathy and IBM, further supporting the hypothesis that the presence of treatment-resistant sarcoidosis is associated with the development or transformation to IBM.

## Case presentation

A 46-year-old African American female with a five-year history of biopsy-confirmed sarcoidosis presented to the clinic with complaints of progressive right upper extremity weakness and bilateral lower extremity weakness accompanied by shortness of breath, headaches, blurred vision, cachexia, and fatigue. Physical examination revealed a waddling gait with heel standing difficulty. There was no evidence of bulbar involvement. Muscle strength testing, using the Medical Research Council (MRC) muscle grading scale, revealed prominent right shoulder abduction weakness (2/5) and symmetrically reduced proximal muscle strength with weakness in shoulder flexion/extension (4/5), elbow flexion/extension (4/5), hip flexion/extension (4/5) and knee flexion/extension (4/5). Upper extremity reflexes were lively (2/4), while lower extremity patellar and ankle jerk reflexes were diminished (1/4).

Serology revealed elevated total creatine kinase (CK) of 574 units/L (normal 24-173 units/L), approximately three times the upper normal limit. Upper extremity electromyography (EMG) revealed early recruitment and myopathic units in the right deltoid, biceps, and supraspinatus. Lower extremity EMG revealed myopathic units involving the tibialis anterior and peroneus longus without evidence of early recruitment. There was no evidence of myopathic units involving the quadriceps. Lower extremity nerve conduction studies were normal. Magnetic resonance imaging of the neuroaxis was unremarkable. Over the course of five years, the patient had received treatment with prednisone, methylprednisolone, and azathioprine with little improvement.

**Figure 1:**
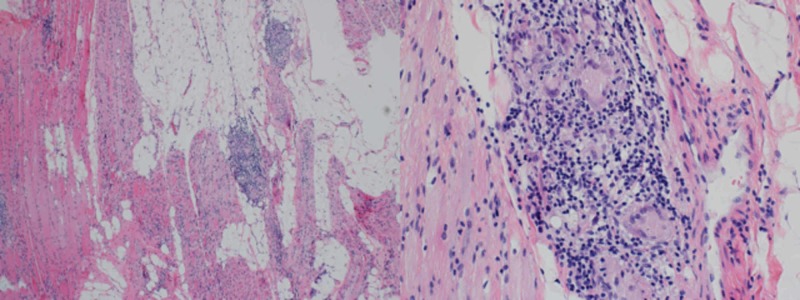
Hematoxylin and eosin stain of a deltoid biopsy revealing the presence of marked muscle atrophy, non-necrotizing granulomatous myositis, and inflammatory infiltrates

One year later, the patient returned to the clinic with progressive muscle weakness. Physical examination revealed a waddling gait, now requiring a cane for ambulation. Worsening motor deficits included bilateral shoulder abduction (2/5) and bilateral hip flexion (2/5). Finger flexor weakness and ulnar atrophy were absent. Based on these findings, the patient was sent for a deltoid muscle biopsy which revealed the presence of granulomatous myositis (Figure 1) consistent with sarcoid myopathy.

**Figure 2:**
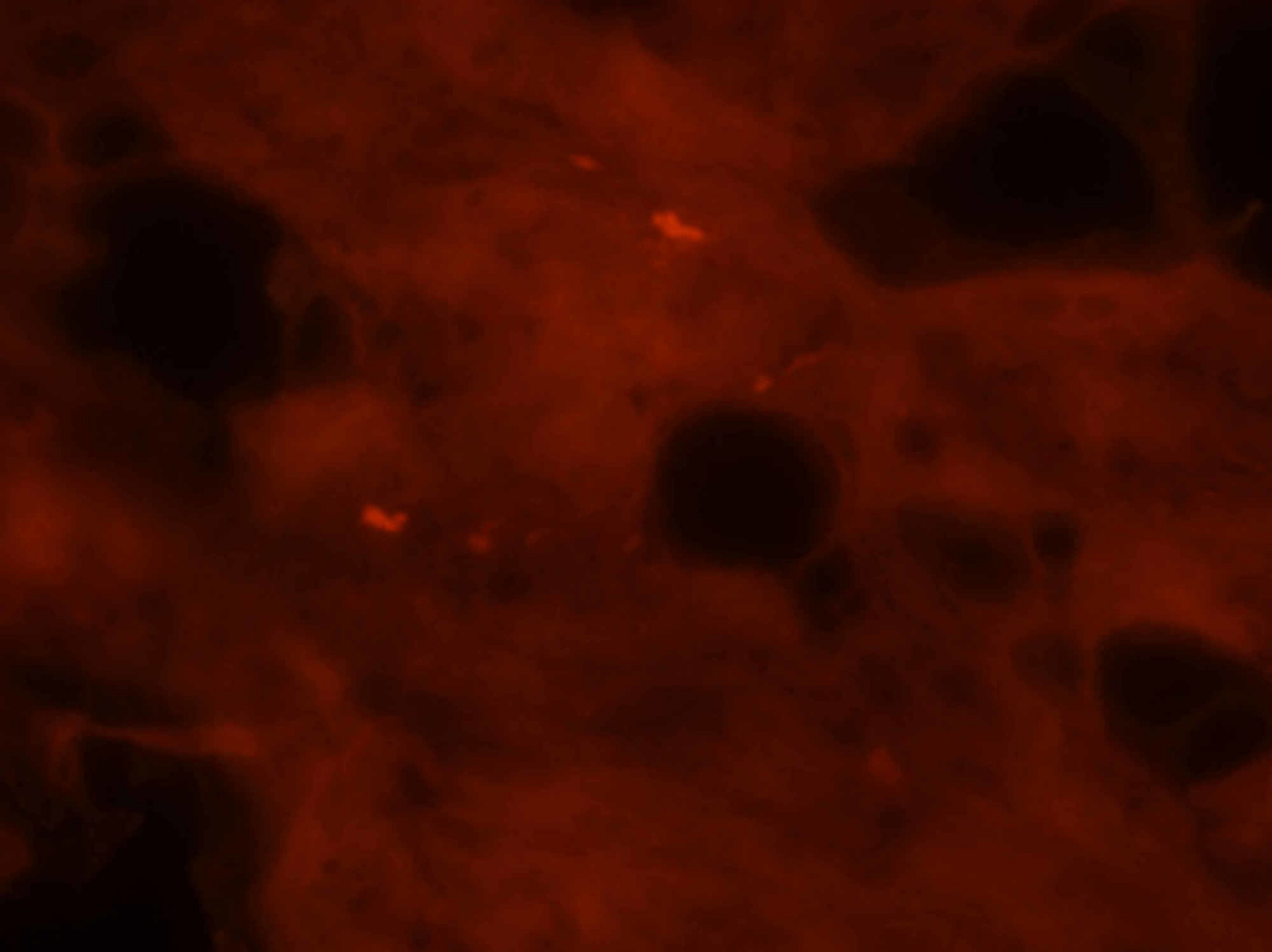
Congo red stain reveals muscle fibers containing congophilic intracellular inclusions

**Figure 3:**
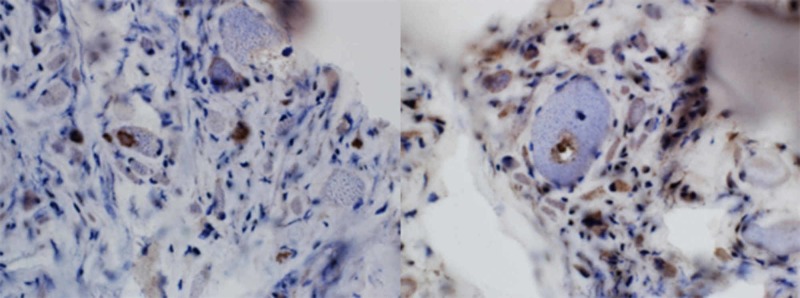
TDP43/P62 staining reveals protein accumulations and a rimmed vacuole

Additional findings included COX-negative muscle fibers accompanied by marked muscle atrophy (Figure 1), congophilic intracellular inclusions (Figure 2), and TDP43/p62-positive rimmed vacuole type structures (Figure 3) consistent with co-existing sporadic inclusion body myositis.

The patient was subsequently initiated on a trial of intravenous infliximab, which failed to improve the patient’s symptoms.

## Discussion

Sarcoidosis is a systemic inflammatory immune disorder disproportionately affecting African Americans [8]. The prevalence of sarcoidosis is estimated at 10-65 cases per 100,000 persons with only five percent of patients experiencing central nervous system involvement [9]. The diagnosis of sarcoidosis relies on clinical and radiological presentation along with histologic or pathologic evidence of noncaseating granulomas in the absence of other diseases [10]. Muscle involvement is rare - estimated at a rate of 0.5-2.5% - and is often asymptomatic [11]. A systemic review and meta-analysis of 1,088 neurosarcoidosis patients showed a mean age of presentation to be 43 with variable outcomes to include remission, the stability of the disease, and in a minority of cases, deterioration or death [11]. Most patients with sarcoidosis respond well to glucocorticoid therapy [12].

Inclusion body myositis (IBM) is a much rare and ominous progressive muscle disorder with a prevalence estimated at 25 cases per million persons [13]. IBM is more common in men than women and typically arises after age 50. Weakness and atrophy of proximal limbs occur earlier in the disease followed by bulbar involvement in later stages [14]. Steady declines in strength range from three to five percent per year, confining many patients to wheel-chairs within years of the initial diagnosis [15]. A triad of physical examination findings is characteristic of IBM and includes weakness in finger flexion, hip flexion, and ankle dorsiflexion. Of these symptoms, finger flexion weakness has been shown to be highly specific to IBM compared to other idiopathic myopathies [16]. Additional clinical indicators of IBM include increased creatine kinase, but less than 15 times the upper normal limit, and abnormal electromyograms with myopathic and neuropathic units. Development of serological assays targeting the purported IBM autoantigen cytosolic 5’-nucleotidase 1A (CN-1A) has been shown to be highly specific and moderately sensitive for diagnosing IBM [17]. However, the mainstay of IBM diagnosis is a muscle biopsy featuring increased surface muscle MHC-1 expression, rimmed vacuoles and intracellular amyloid deposits [18]. Unlike sarcoidosis, there are no effective treatment options available for IBM.

A biopsy is an invaluable tool in the diagnosis of both IBM and sarcoid myopathy. In patients with worsening symptoms and poor treatment outcomes, subsequent biopsies may prove beneficial in elucidating underlying pathology. Unlike sarcoidosis, glucocorticoids and immunosuppressive treatments do not alter the natural course of IBM. In fact, a long-term observational study revealed that early immunosuppressant drug therapy could modestly exacerbate the progression of debilitation in IBM patients [2]. This clinical overlap between sarcoidosis and IBM creates a dilemma by which a clinician may treat sarcoidosis while exacerbating the symptoms of IBM. Fortunately, trials aimed at therapeutically targeting symptomatic atrophic processes via myostatin (which prevents muscle building) have shown improvement in muscle volume and walking time for patients with IBM [19]. While the results of the trials are promising, larger studies are required before treatment conclusions can be drawn.

Co-existing sarcoidosis and IBM has been documented infrequently since the first instance in 1986 [7]. While the pathogenesis of these diseases has yet to be discovered, contemporary theories point to the shared involvement of MHC-1 signaling common to both diseases [20]. In this case, the patient transitioned from responding to treatment with milder symptoms (attributed to sarcoidosis) to becoming refractory to treatment with more severe symptoms (attributed to IBM). This disease progression, accompanied by an indirect correlation to treatment efficacy, suggests an interplay between sarcoidosis and IBM pathogenesis. This case highlights the importance of monitoring patients with sarcoidosis for unusual disease progression. Further research is needed into the progression of co-existing disease populations to determine the relationship between these diseases.

## Conclusions

Sarcoid myopathy and inclusion body myositis are idiopathic inflammatory diseases with overlapping symptoms and contrasting treatment strategies. The co-existence of both diseases has rarely been reported. This case provides further support to an association between sarcoid myopathy and inclusion body myositis pathogenesis. Additionally, it calls for further research into the progression and treatment of both diseases, since they may represent a complex inflammatory continuum.
